# Diadochic single crystal of an erbium–neodymium nitrate complex

**DOI:** 10.1107/S2056989026004615

**Published:** 2026-05-12

**Authors:** Vinh T. Nguyen, Jarrod M. Gogolski, Matthew S. Mills, Binod K. Rai

**Affiliations:** a Savannah River National Laboratory (SRNL) Aiken SC USA; University of Missouri-Columbia, USA

**Keywords:** neodymium, erbium, single-crystal, substitution

## Abstract

The synthesis and characterization of a single-crystal of tetra­aqua­tri­nitratoerbium/neodymium(0.359/0.641) dihydrate, Er_0.359_Nd_0.641_(NO_3_)_3_(H_2_O)_4_·2H_2_O, is reported herein. X-ray diffraction highlighted a crystal with structural features, including a network of water mol­ecules and extensive hydrogen bonding between layers, that are isomorphous with other light lanthanide crystal structures. Single-crystal XRD and SEM-EDS indicated the substitutional behavior of a heavy lanthanide, Er, into the lattice site of a light lanthanide, Nd. The diadochic nature of the lanthanides within a nitrate-containing structure culminated in a 1:0.95 ratio of Er:Nd.

## Abstract 2

Tetra­aqua­trinitratoerbium/neodymium(0.359/0.641) dihydrate exhibits structural features, including a network of water mol­ecules and extensive hydrogen bonding between layers, that are isomorphous with other light lanthanide crystal structures.

## 1. Chemical context

Since their discovery, lanthanides have become increasingly prominent in numerous fields of research, including biomed­ical (Martinez-Gomez *et al.*, 2016 ▸; Cotruvo, 2019 ▸; Eliseeva & Bünzli, 2010 ▸), energy (Zepf *et al.*, 2014 ▸; De Jesus *et al.*, 2021 ▸), and other modern technological fields (Tessitore *et al.*, 2023 ▸). Advances in these fields have increased global demand for lanthanides (Dutta *et al.*, 2016 ▸). Though global reserves indicate rare-earth elements, which include lanthanides, are abundant, major natural sources are limited to few geographical locations with fewer commercial suppliers. Since the majority of rare-earth metals are processed in China, a disruption in supply chains could significantly impact research and production (Tukker, 2014 ▸). Technologies to improve the separation, purification, and recovery of lanthanides could help alleviate future supply bottlenecks and dependency on near-monopolistic suppliers (Sinha *et al.*, 2016 ▸; Omodara *et al.*, 2019 ▸; Izatt *et al.*, 2014 ▸). Aqueous processing in nitric acid media is the conventional route for the large-scale separation of lanthanides (Xie *et al.*, 2014 ▸). The behavior of lanthanides, particularly during crystallization or precipitation, is of key inter­est during their recovery and processing (Yatsenko *et al.*, 2021 ▸; Li *et al.*, 2024 ▸). For mixtures containing impurities or multiple lanthanides, similarly sized elements can substitute for one another within a crystal structure. These substitutional impurities can result in diadochic crystals, or substituted crystals. Isomorphic structures typically observed with light lanthanides could incorporate with heavy lanthanides (Leskelä *et al.*, 1986 ▸). Identifying these diadochic structures enhances lanthanide recovery processes through a better understanding of their coprecipitation behavior and analytical signatures. Herein, we report the characterization of an Er (heavy lanthanide) substitution into an Nd (light lan­thanide) nitrate crystal with water ligands, [(Er/Nd)(NO_3_)_3_(H_2_O)_4_]·2H_2_O.

## 2. Structural commentary

**Figure 1:**
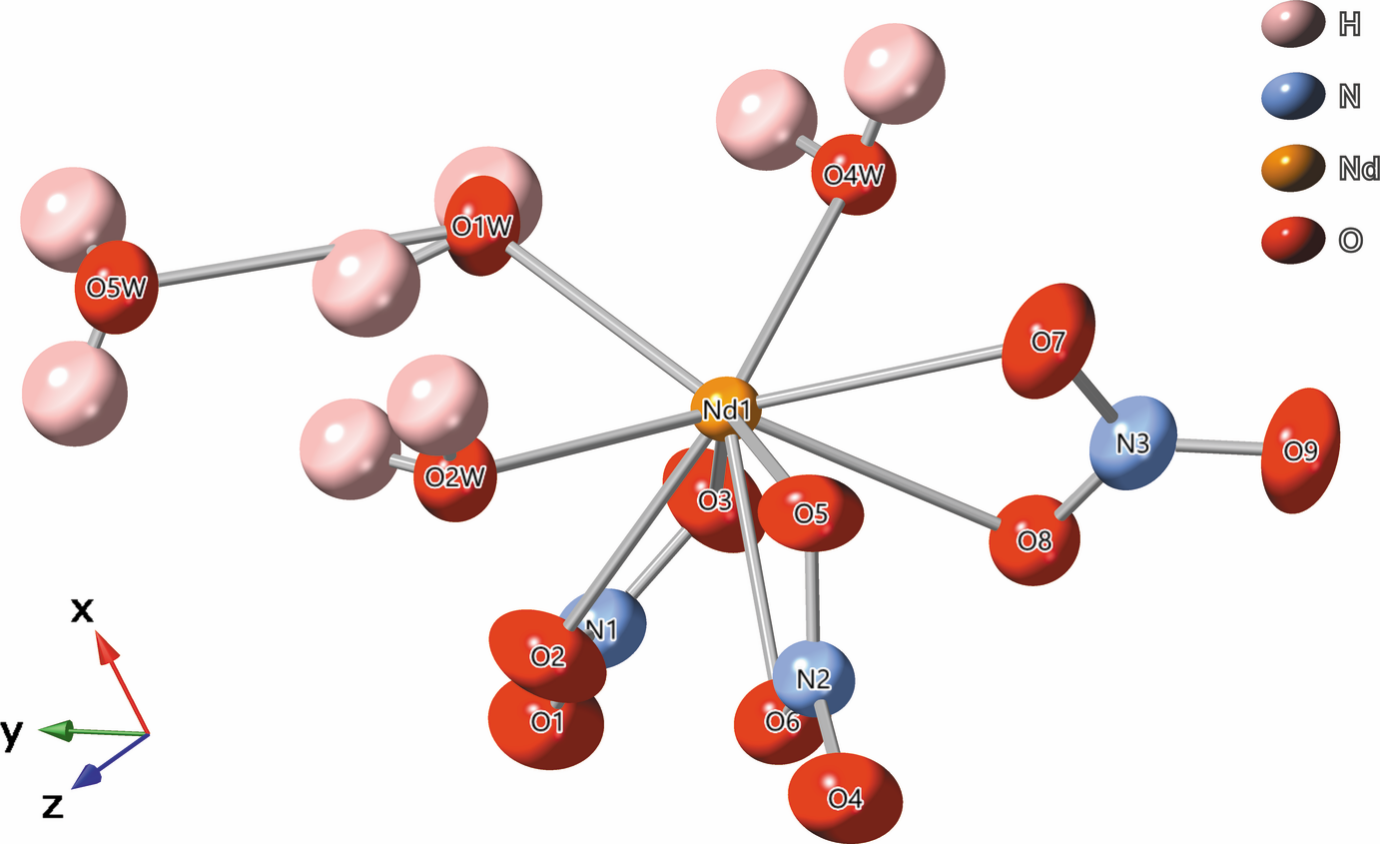
Visual representation (50% ellipsoids) of the local environment around the central metal atom.

Single-crystal X-ray Diffraction (XRD) analysis revealed a triclinic cell with space group *P*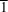, density of 2.370 g cm^−3^, and an empirical formula of Er_0.359_Nd_0.641_H_12_N_3_O_15_ (Fig. 1 ▸). Presuming an Er/Nd substitution, the formula could be arranged as [Er_0.359_Nd_0.641_(NO_3_)_3_(H_2_O)_4_]·2H_2_O.

**Figure 2:**
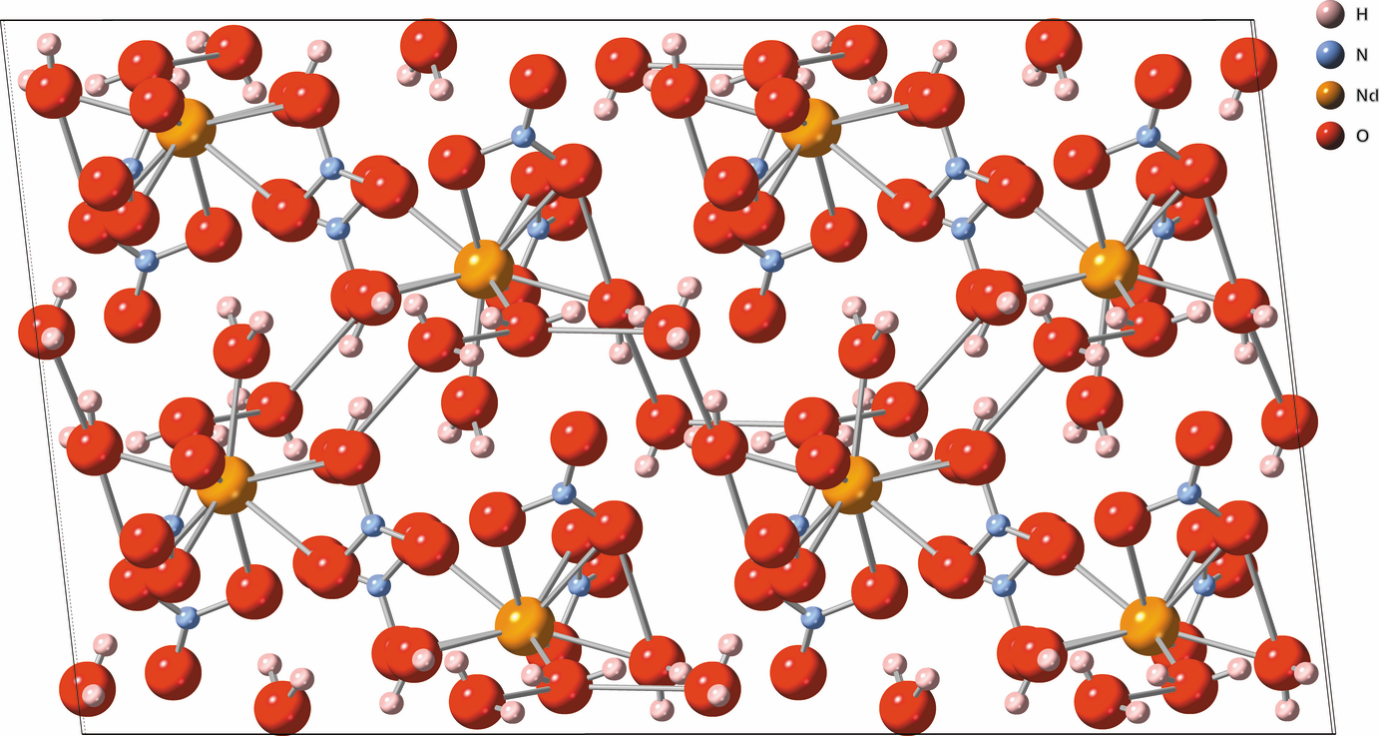
Visual representation viewed along the *b*-axis direction.

The refined crystal structure suggests that the structure is diadochic in nature, with the central metal atom, *M*, being either Er or Nd. Coordinated to this central Er or Nd center are ten oxygen atoms. Four of these oxygen atoms belong to water mol­ecules while the remaining six oxygen atoms belong to three bidentate nitrate ligands. The bond lengths between the metal center and the chelating oxygen atoms (O2, O3, O5, O6, O7, O8) of the nitrate ligands range between 2.488 (2)–2.765 (3) Å (Fig. 1 ▸). Several of these *M*—O bond lengths [2.555 (2), 2.577 (13), and 2.765 (3) Å] are rather elongated and are outside the estimated sum of the covalent radii of Er—O (2.55 Å) or Nd—O (2.67 Å) radii (Cordero *et al.*, 2008 ▸). The chemical formula indicates that a total of six water mol­ecules are part of the crystal structure. Of these six water mol­ecules, four equivalents of water (O1*W*, O2*W*, O3*W*, O4*W*) are coordinated to the metal center with *M*—O bond distances ranging from 2.375 (2)–2.412 (2) Å. Two water mol­ecules (O5*W* and O6*W*) are not coordinated to the metal centers, but belong to the crystal structure as part of a network of hydrogen bonds (Table 1 ▸, Fig. 2 ▸). The O5*W* water mol­ecule bridges the O1*W* and O2*W* water mol­ecules while the O6*W* water mol­ecule bridges the O1*W* and O4*W* water mol­ecules. This structure is isomorphous with other light lanthanide structures (Decadt *et al.*, 2012 ▸; Gshneider & Eyring, 1986 ▸; Kawashima *et al.*, 2000 ▸; Rogers *et al.*, 1983 ▸; Shi & Wang, 1991 ▸; Stumpf & Bolte, 2001 ▸).

## 3. Supra­molecular features

**Figure 3:**
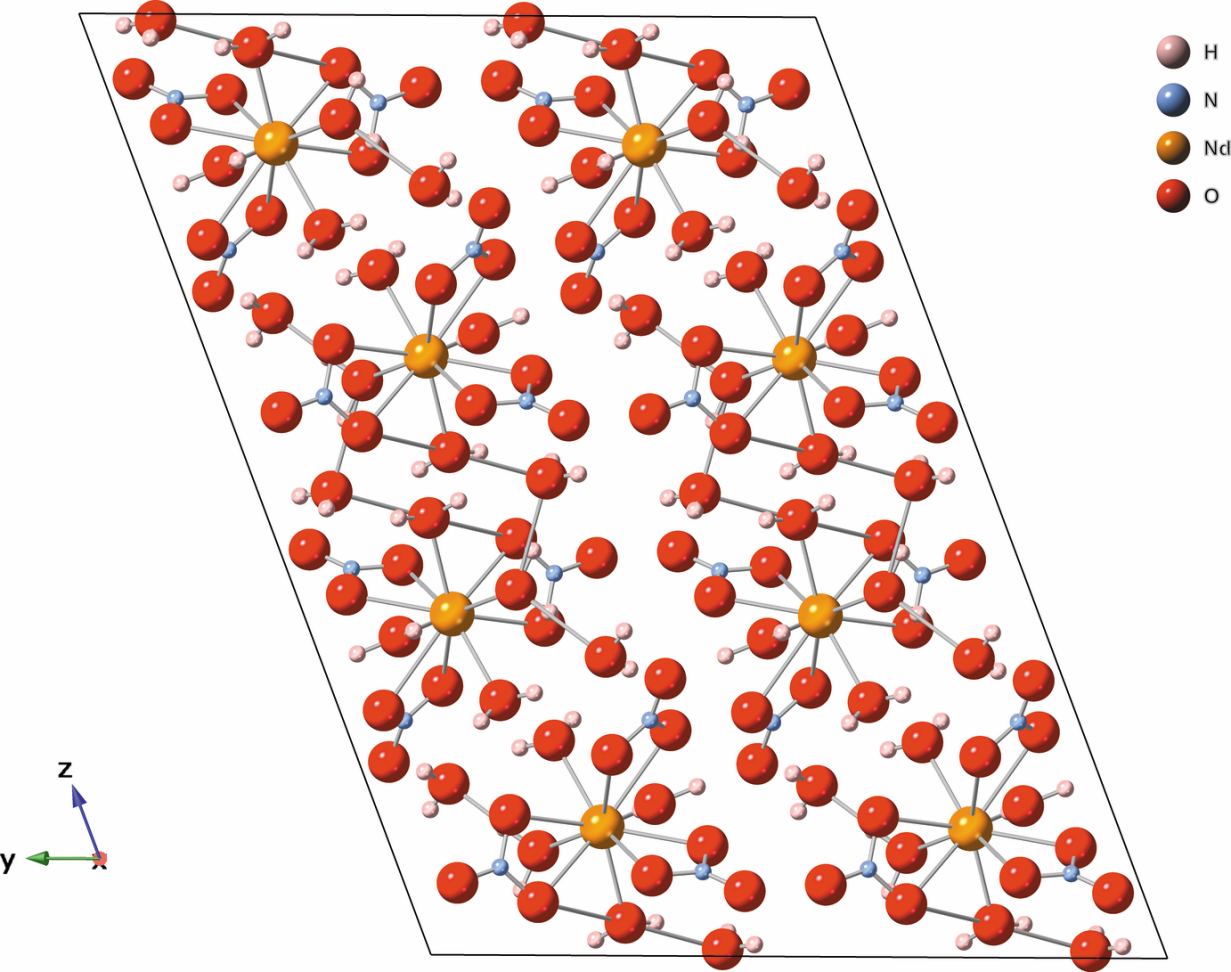
Visual representation viewed along the *a*-axis direction.

**Figure 5:**
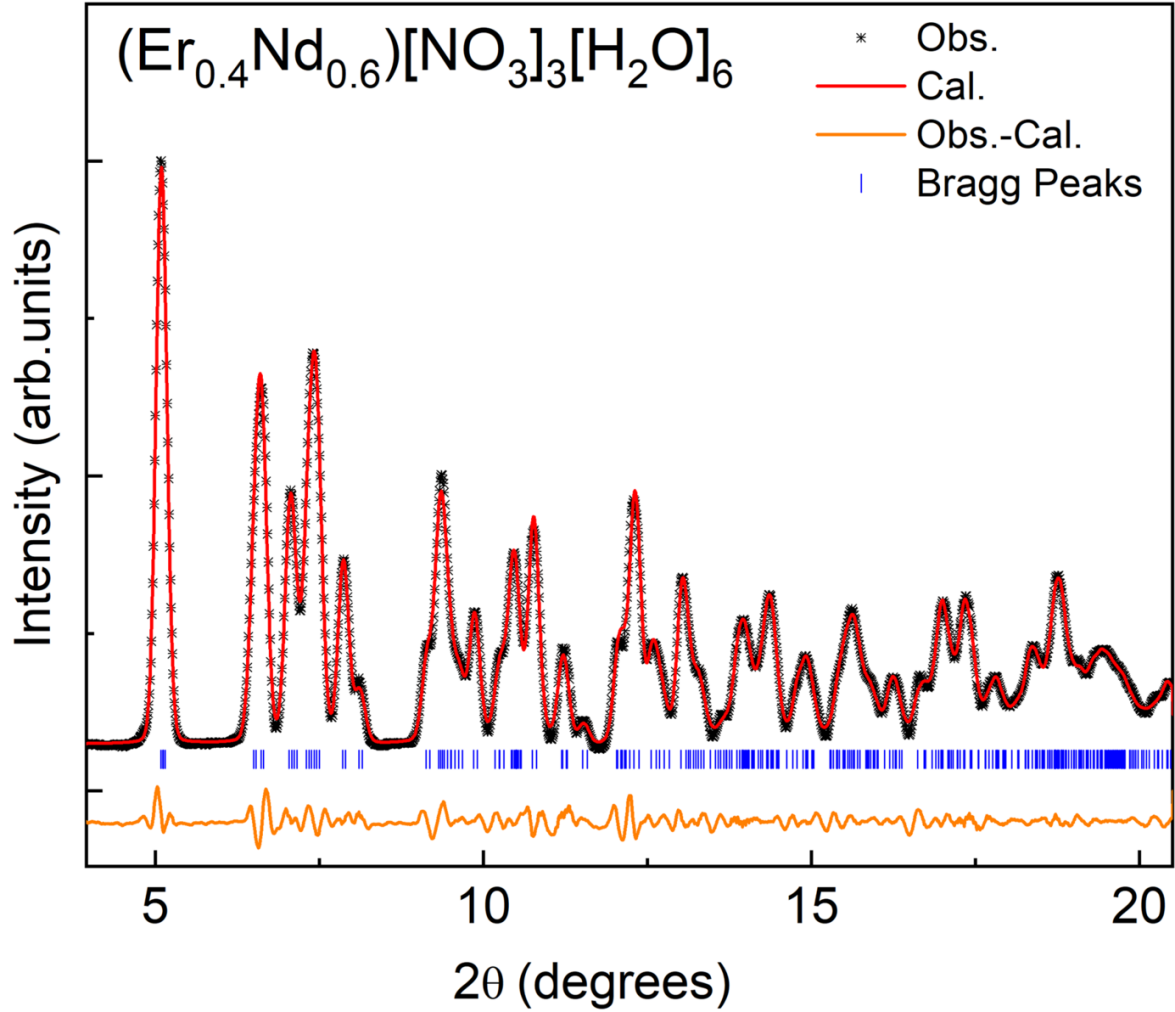
Profile matching of powder XRD of the title compound at room temperature using *FullProf* (Rodríguez-Carvajal, 1993 ▸).

When viewed along crystallographic *a*-axis direction, the crystal appears to have layers that are far enough apart to suggest there are no distinct chemical bonds between the layers (Figs. 3 ▸ and 4 ▸). However, the inter­face between each layer contains nitrate anions and water mol­ecules, and the distances between the water mol­ecules and nitrate oxygen atoms are below 3 Å (Table 1 ▸), which is within the range for hydrogen bonding between the layers (Shen *et al.*, 1990 ▸). Extensive networks of hydrogen bonds between water mol­ecules and nitrate ions have been documented for lanthanide compounds (Yatsenko *et al.*, 2021 ▸). Greater degrees of hydrogen bonding support charge transfer between the water mol­ecules, lanthanide, and nitrate anions that increases the overall stability (Yatsenko *et al.*, 2021 ▸). The phase purity and crystal quality of [Er_0.359_Nd_0.641_(NO_3_)_3_(H_2_O)_4_]·2H_2_O were confirmed at room temperature using Rietveld refinement (see Fig. 5 ▸).

## 4. Scanning Electron Microscopy-Energy Dispersive X-ray Spectroscopy

**Figure 6:**
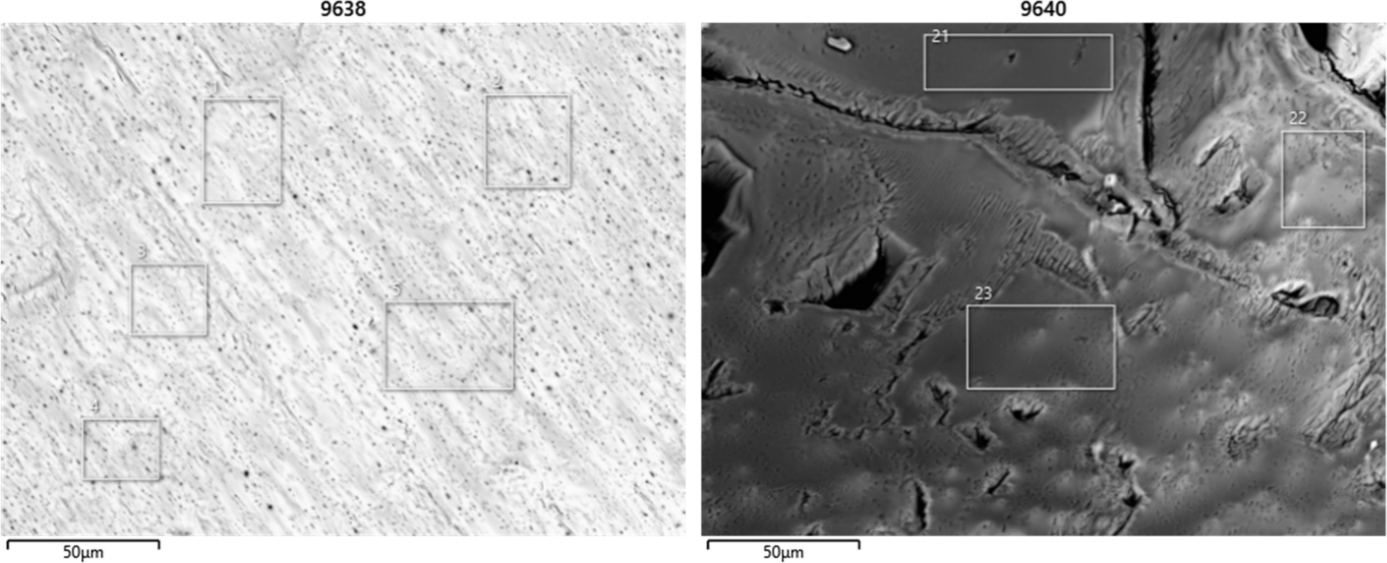
SEM-EDS images of an unpolished crystal.

**Figure 7:**
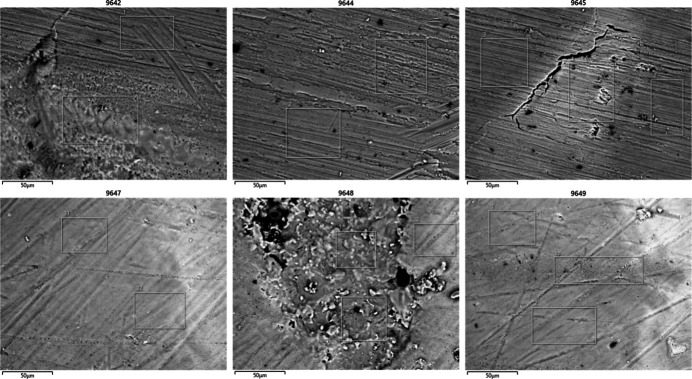
SEM-EDS images of a crystal polished by sanding.

To assess the partial substitution of Er into the Nd site, scanning electron microscope (SEM) images were taken and analyzed with energy dispersive X-ray spectroscopy (EDS). Since crystal surfaces coated with residual Er or Nd from the recrystallization process would not be representative of the crystal composition, two types of samples were collected. Crystals were either analyzed without modification (Fig. 6 ▸), or were polished by sanding prior to analysis (Fig. 7 ▸). Presumably, sanding the crystals removed the surface layers of the crystals and exposed the inter­ior. Selected cross sections of both the unpolished and polished crystals were analyzed by EDS. The Er and Nd percent compositions of the selected cross sections are tabulated in Tables 2 ▸ and 3 ▸. The remaining elemental percentage compositions are comprised of C, O, or N, and are omitted for clarity. Variations in sample morphology or matrices rendered the SEM-EDS analysis as qualitative. Comparing the Er and Nd composition of both the unpolished crystal and sanded crystals revealed an average Er:Nd ratio of 1.89 (37) and 0.95 (58), respectively. The error was calculated by determining the standard deviation of the measured SEM-EDS values. The average values indicated the unpolished crystal surfaces had approximately twice the Er content than the inter­ior of the crystals, with minimal overlap between the ratios when accounting for standard deviation. A comparison of these two ratios suggested the surface layer of the crystal was coated in precipitates with elemental compositions that did not accurately represent that of the crystal structure.

## 5. Database survey

A search of the Cambridge Structural Database (CSD Version 6.00, updated May 2025) yielded two notable Er and Nd crystal structures containing both nitrate anions and water mol­ecules. The first structure was published by Klein and has the chemical formula, H_10_ErN_3_O_14_ or [Er(NO_3_)_3_(H_2_O)_4_]·H_2_O (Klein, 2022 ▸). Though this structure has one equivalent of water fewer than the structure reported herein, Klein’s structure also contains three bidentate nitrate ligands and four water mol­ecules coordinated to the central Er atom. The fifth and final water mol­ecule is not bound to Er. Furthermore, the geometry of Klein’s structure differs from the structure reported herein. The coordinated nitrate ions in Klein’s structure could be loosely described as ‘pseudo-meridional’, with a plane drawn between the nitro­gen atoms roughly bis­ecting an imagined sphere about the metal center. In contrast, the nitrate ligands on the reported [Er_0.359_Nd_0.641_(NO_3_)_3_(H_2_O)_4_]·2H_2_O crystal structure are oriented towards one face of an envisioned polygon drawn from the metal’s coordination sphere.

Crystal structures, reported by Rogers, Shi and co-workers, have been identified with the empirical formula, H_12_N_3_NdO_15_ or [Nd(NO_3_)_3_(H_2_O)_4_]·2H_2_O (Rogers *et al.*, 1983 ▸; Shi & Wang, 1991 ▸). Accounting for cell settings, lattice constants from Shi (*a* = 6.7768, *b* = 9.195, *c* = 11.726 Å) and Rogers (*a* = 9.307, *b* = 11.747, *c* = 6.776 Å) resemble our reported structure, *viz. a* = 6.7423 (1), *b* = 9.1281 (2), *c* = 11.6431 (2) Å. Furthermore, the Nd atom is coordinated to the same number of water mol­ecules and nitrate ligands, which have a similar denticity and geometry about the metal center. The lattice parameters of [Er_0.359_Nd_0.641_(NO_3_)_3_(H_2_O)_4_]·2H_2_O are slightly smaller than those of Nd(NO_3_)_3_·H_2_O. Isomorphous structures have been reported with other lanthanides (Wickleder, 2002 ▸), but an equivalent structure has not been reported with Er.

Refinement of our reported crystal, with Er and Nd refined separately, presents an opportunity for comparison with other isomorphic structures. The average bond lengths between various lanthanide centers and their coordinating atoms illustrate the lanthanide contraction effect, with heavier lanthanides having relatively contracted bond distances (Decadt *et al.*, 2012 ▸; Kawashima *et al.*, 2000 ▸; Shi & Wang, 1991 ▸; Stumpf & Bolte, 2001 ▸; Taha *et al.*, 2012 ▸) are listed in Table 4 ▸. When comparing the average Nd bond lengths of reported structures with our refined Nd structure, the average bond length of our reported structure is shorter. Presumably, substitution with Er, a heavier lanthanide, perturbed the structure towards one with shorter lanthanide bond lengths.

## 6. Synthesis and crystallization

Er(NO_3_)_3_·5H_2_O (99.9%) and Nd(NO_3_)_3_·6H_2_O (99.9%) were purchased from Sigma Aldrich. A 0.723 g (1.65 mmol) sample of Nd from Nd(NO_3_)_3_·6H_2_O and a 0.818 g (1.85 mmol) sample of Er from Er(NO_3_)_3_·5H_2_O was dissolved in 7.41 mL of 8 *M* nitric acid. The solution was agitated to ensure complete dissolution of the solids and then diluted to 25 mL with deionized water. The solution was then air-sparged to dryness. Purple single crystals were collected, and rapidly encased in ep­oxy, to determine their room-temperature crystal structure. It was later found that the crystals deliquesce at room temperature, illustrated in Figs. S1 and S2. Powder XRD data were obtained using the powder diffraction option of the single-crystal XRD instrument and utilizing the *FullProf* program (Rodríguez-Carvajal, 1993 ▸). SEM-EDS data collection was conducted on a Carl Zeiss Microscopy LLC Sigma VP field emission SEM with secondary electron, backscattered electron, and in-lens secondary electron detectors. This instrument has the variable pressure option, which allows a variable pressure up to 133 Pa of nitro­gen gas to reduce or eliminate charging for uncoated samples. EDS was performed using an Oxford Instruments X-Max 20 silicon drift detector to detect elements.

## 7. Refinement

Crystal data, data collection and structure refinement details are summarized in Table 5 ▸. H atoms were refined with *U*_iso_(H) = 1.5*U*_eq_(O).

## Supplementary Material

Crystal structure: contains datablock(s) I. DOI: 10.1107/S2056989026004615/ev2024sup1.cif

Structure factors: contains datablock(s) I. DOI: 10.1107/S2056989026004615/ev2024Isup3.hkl

Supplemental Figure S1. Microscope image of deliquescent solids. DOI: 10.1107/S2056989026004615/ev2024sup4.jpg

Microscope image of deliquesced sample. DOI: 10.1107/S2056989026004615/ev2024sup5.jpg

Supplementary cif with Er-Nd refined. DOI: 10.1107/S2056989026004615/ev2024sup6.txt

CCDC reference: 2551502

Additional supporting information:  crystallographic information; 3D view; checkCIF report

## Figures and Tables

**Figure 4:**
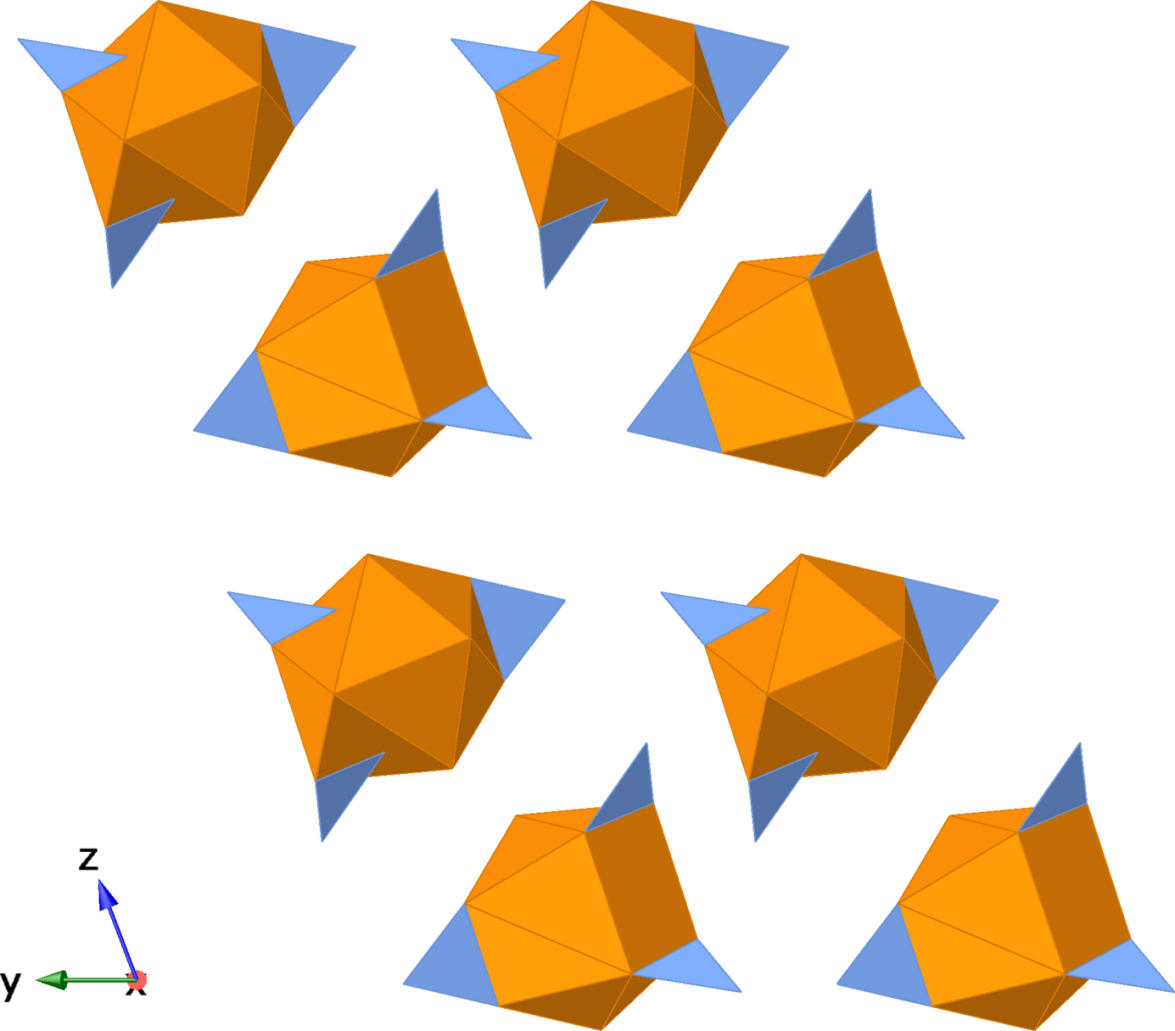
Visual representation (polyhedral) viewed along the *a*-axis direction.

**Table 1:** Hydrogen-bond geometry (Å, °)

*D*—H⋯*A*	*D*—H	H⋯*A*	*D*⋯*A*	*D*—H⋯*A*
O1*W*—H1*WA*⋯O6*W*^i^	0.99	1.84	2.744 (4)	150
O1*W*—H1*WB*⋯O5*W*^ii^	0.94	1.87	2.743 (4)	153
O2*W*—H2*WA*⋯O5*W*	0.94	1.79	2.678 (4)	155
O2*W*—H2*WB*⋯O5*W*^ii^	0.91	2.39	3.274 (4)	163
O2*W*—H2*WB*⋯O6^iii^	0.91	2.58	2.976 (4)	107
O3*W*—H3*WA*⋯O4^iv^	1.06	2.18	3.076 (4)	140
O3*W*—H3*WA*⋯O5^iv^	1.06	2.15	3.095 (4)	146
O3*W*—H3*WB*⋯O6^v^	1.03	2.35	3.206 (4)	140
O3*W*—H3*WB*⋯O8^v^	1.03	2.35	3.204 (4)	139
O4*W*—H4*WA*⋯O6*W*	0.93	1.85	2.727 (4)	156
O4*W*—H4*WB*⋯O6*W*^i^	0.94	2.39	3.312 (4)	166
O5*W*—H5*WA*⋯O4^v^	0.85	2.05	2.849 (4)	156
O5*W*—H5*WB*⋯O1^iii^	0.85	2.38	3.032 (4)	134
O5*W*—H5*WB*⋯O7^iv^	0.85	2.27	2.897 (4)	130
O6*W*—H6*WA*⋯O9^v^	0.85	1.99	2.834 (5)	176
O6*W*—H6*WB*⋯O1^vi^	0.85	2.19	2.922 (4)	144

Symmetry codes: (i) 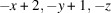; (ii) 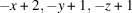; (iii) 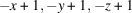; (iv) 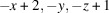; (v) 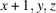; (vi) 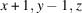.

**Table 2:** Percentage of Nd and Er and the Er:Nd ratio for several EDS-examined cross sections of unpolished crystal surfaces

SEM image No.	Cross section No.	%Nd	%Er	Er:Nd ratio
9638	1	17.62	28.65	1.63
9638	2	18.84	29.96	1.59
9638	3	15.99	30.12	1.88
9638	4	18.19	26.04	1.43
9638	5	17.89	31.15	1.74
9640	21	5.79	14.44	2.49
9640	22	14.45	32.52	2.25
9640	23	16.44	35.08	2.13
Average				1.89

**Table 3:** Percentage of Nd and Er and the Er:Nd ratio for several EDS-examined cross sections of sand-polished crystal surfaces

SEM image No.	Cross section No./description	%Nd	%Er	Er:Nd ratio
9642	1	21.09	24.62	1.17
9642	2	16.88	24.69	2.14
9644	1	35.04	35.86	1.02
9644	2	21.81	21.53	0.99
9645	1	29.72	37.39	1.26
9645	2	31.07	37.71	1.21
9645	3	17.18	32.42	1.89
9647	31	24.75	7.28	0.29
9647	32	23.65	7.02	0.30
9648	1	19.23	23.46	1.22
9648	2	23.83	10.10	0.42
9648	3	16.10	22.67	1.41
9649	Flat area 1	25.33	7.00	0.28
9649	Flat area 2	16.10	22.67	1.41
9649	Bright spot	3.35	2.40	0.72
9649	Spongy area	37.16	21.42	0.58
Average				0.95

**Table 4:** Bond lengths (Å) between the central lanthanide atom and the coordinating atom of various reported isomorphous structures^*a*^

Atom	Er^*b*^	Nd^*b*^	Pr	Nd	Sm	Eu	Gd
O2	2.5168	2.4716	2.5677	2.5492	2.5473	2.5104	2.5282
O3	2.6170	2.4776	2.5790	2.5609	2.5155	2.5367	2.4940
O5	2.7170	2.7977	2.7306	2.7125	2.5383	2.7405	2.5177
O6	2.5659	2.5503	2.6155	2.6008	2.6003	2.5673	2.5783
O7	2.5536	2.5005	2.5999	2.5688	2.7061	2.5393	2.7537
O8	2.6730	2.5136	2.6346	2.6155	2.5785	2.5889	2.5517
O1*W*	2.3368	2.4055	2.4289	2.4432	2.3962	2.3788	2.3639
O2*W*	2.3137	2.4613	2.4470	2.4496	2.4330	2.4027	2.3977
O3*W*	2.3234	2.4543	2.4580	2.4577	2.4275	2.4062	2.3919
O4*W*	2.4615	2.3823	2.4556	2.4615	2.4233	2.4118	2.3891
Average	2.5079	2.5015	2.5517	2.5420	2.5166	2.5083	2.4966

Notes: (*a*) (Decadt *et al.*, 2012 ▸; Kawashima *et al.*, 2000 ▸; Shi & Wang, 1991 ▸; Stumpf & Bolte, 2001 ▸; Taha *et al.*, 2012 ▸); (*b*) values obtained from reported structures with Er and Nd refined separately.

**Table 5:** Experimental details

Crystal data	
Chemical formula	Er_0.359_Nd_0.641_(NO_3_)_3_(H_2_O)_4_·2H_2_O
*M*_r_	446.65
Crystal system, space group	Triclinic, *P* 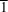
Temperature (K)	298
*a*, *b*, *c* (Å)	6.7423 (1), 9.1281 (2), 11.6431 (2)
α, β, γ (°)	70.197 (2), 88.881 (1), 69.165 (2)
*V* (Å^3^)	625.94 (2)
*Z*	2
Radiation type	Mo *K*α
μ (mm^−1^)	5.16
Crystal size (mm)	0.26 × 0.20 × 0.17

Data collection	
Diffractometer	XtaLab Synergy
Absorption correction	Multi-scan (*CrysAlis PRO*; Rigaku OD, 2023 ▸)
*T*_min_, *T*_max_	0.935, 1.000
No. of measured, independent and observed [*I* > 2σ(*I*)] reflections	16864, 3861, 3468
*R*_int_	0.042
(sin θ/λ)_max_ (Å^−1^)	0.725

Refinement	
*R*[*F*^2^ > 2σ(*F*^2^)], *wR*(*F*^2^), *S*	0.028, 0.060, 1.04
No. of reflections	3861
No. of parameters	184
H-atom treatment	H-atom parameters constrained
Δρ_max_, Δρ_min_ (e Å^−3^)	0.78, −0.85

Computer programs: *CrysAlis PRO* (Rigaku OD, 2023 ▸), *SHELXT2018/2* (Sheldrick, 2015*a* ▸), *SHELXL2018/3* (Sheldrick, 2015*b* ▸) and *OLEX2* (Dolomanov *et al.*, 2009 ▸).

## supplementary crystallographic information

### Tetraaquatrinitratoerbium/neodymium(0.359/0.641) dihydrate Crystal data

**Table:**

Er_0.359_Nd_0.641_(NO_3_)_3_(H_2_O)_4_·2H_2_O	*Z* = 2
*M**_r_* = 446.65	*F*(000) = 432
Triclinic, *P*1	*D*_x_ = 2.370 Mg m^−^^3^
*a* = 6.7423 (1) Å	Mo *K*α radiation, λ = 0.71073 Å
*b* = 9.1281 (2) Å	Cell parameters from 10752 reflections
*c* = 11.6431 (2) Å	θ = 2.5–30.8°
α = 70.197 (2)°	µ = 5.16 mm^−^^1^
β = 88.881 (1)°	*T* = 298 K
γ = 69.165 (2)°	Irregular, purple
*V* = 625.94 (2) Å^3^	0.26 × 0.20 × 0.17 mm

### Tetraaquatrinitratoerbium/neodymium(0.359/0.641) dihydrate Data collection

**Table:**

XtaLab Synergy diffractometer	3861 independent reflections
Radiation source: micro-focus sealed X-ray tube	3468 reflections with *I* > 2σ(*I*)
Detector resolution: 10 pixels mm^-1^	*R*_int_ = 0.042
ω scans	θ_max_ = 31.0°, θ_min_ = 2.6°
Absorption correction: multi-scan (CrysAlis Pro; Rigaku OD, 2023)	*h* = −9→9
*T*_min_ = 0.935, *T*_max_ = 1.000	*k* = −12→13
16864 measured reflections	*l* = −16→16

### Tetraaquatrinitratoerbium/neodymium(0.359/0.641) dihydrate Refinement

**Table:**

Refinement on *F*^2^	Hydrogen site location: difference Fourier map
Least-squares matrix: full	H-atom parameters constrained
*R*[*F*^2^ > 2σ(*F*^2^)] = 0.028	*w* = 1/[σ^2^(*F*_o_^2^) + (0.0238*P*)^2^] where *P* = (*F*_o_^2^ + 2*F*_c_^2^)/3
*wR*(*F*^2^) = 0.060	(Δ/σ)_max_ = 0.001
*S* = 1.04	Δρ_max_ = 0.78 e Å^−^^3^
3861 reflections	Δρ_min_ = −0.85 e Å^−^^3^
184 parameters	Extinction correction: SHELXL2018/3 (Sheldrick 2015b), Fc^*^=kFc[1+0.001xFc^2^λ^3^/sin(2θ)]^-1/4^
0 restraints	Extinction coefficient: 0.0066 (6)

### Tetraaquatrinitratoerbium/neodymium(0.359/0.641) dihydrate Special details

**Table:**

Experimental. The XRD data collection was done using a Rigaku XtaLAB Synergy-S X-ray diffractometer equipped with a HyPix 3000-pixel array detector and a microfocus sealed tube (Mo—Kα radiation at a wavelength of 0.71073 Å, operating at 50 kV and 1 mA). To ensure completeness and desired redundancy, the CrysAlisPro program was used for data collection strategy, data collection, and processing (CrysAlis Pro, 2023). The crystal structure was solved using intrinsic phasing methods with ShelXT and refined with ShelXL through the Olex2 graphical user interface (Sheldrick, 2015a; Sheldrick, 2015b; Dolomanov *et al.*, 2009).
Geometry. All esds (except the esd in the dihedral angle between two l.s. planes) are estimated using the full covariance matrix. The cell esds are taken into account individually in the estimation of esds in distances, angles and torsion angles; correlations between esds in cell parameters are only used when they are defined by crystal symmetry. An approximate (isotropic) treatment of cell esds is used for estimating esds involving l.s. planes.

### Tetraaquatrinitratoerbium/neodymium(0.359/0.641) dihydrate Fractional atomic coordinates and isotropic or equivalent isotropic displacement parameters (Å^2^)

**Table:**

	*x*	*y*	*z*	*U*_iso_*/*U*_eq_	Occ. (<1)
Nd1	0.69801 (2)	0.40409 (2)	0.27428 (2)	0.02539 (7)	0.641 (11)
Er1	0.69801 (2)	0.40409 (2)	0.27428 (2)	0.02539 (7)	0.359 (11)
N1	0.3256 (5)	0.7230 (4)	0.1870 (3)	0.0389 (7)	
N2	0.5844 (5)	0.1654 (4)	0.5018 (3)	0.0356 (7)	
N3	0.5830 (5)	0.1757 (4)	0.1798 (3)	0.0423 (7)	
O1	0.1723 (4)	0.8548 (3)	0.1533 (3)	0.0557 (8)	
O2	0.3986 (4)	0.6435 (3)	0.2986 (2)	0.0534 (8)	
O3	0.4228 (4)	0.6552 (3)	0.1132 (2)	0.0551 (8)	
O4	0.5212 (5)	0.0817 (3)	0.5901 (2)	0.0549 (8)	
O5	0.7736 (4)	0.1198 (3)	0.4805 (3)	0.0542 (8)	
O6	0.4558 (4)	0.3043 (3)	0.4277 (2)	0.0431 (6)	
O7	0.7620 (4)	0.1371 (3)	0.2344 (3)	0.0549 (8)	
O8	0.4450 (4)	0.3185 (3)	0.1707 (2)	0.0470 (7)	
O9	0.5414 (5)	0.0818 (4)	0.1388 (3)	0.0595 (8)	
O1W	0.8666 (4)	0.6027 (3)	0.2219 (2)	0.0443 (6)	
H1WA	0.817420	0.688578	0.139041	0.066*	
H1WB	0.834866	0.671754	0.269792	0.066*	
O2W	0.7787 (4)	0.4471 (3)	0.4575 (2)	0.0384 (6)	
H2WA	0.913068	0.374744	0.502452	0.058*	
H2WB	0.792410	0.547515	0.441551	0.058*	
O3W	1.0723 (3)	0.2377 (3)	0.3232 (2)	0.0430 (7)	
H3WA	1.154156	0.105318	0.360449	0.065*	
H3WB	1.194735	0.282579	0.310698	0.065*	
O4W	0.8012 (4)	0.4387 (3)	0.0701 (2)	0.0390 (6)	
H4WA	0.932392	0.355940	0.070968	0.058*	
H4WB	0.830059	0.536859	0.033308	0.058*	
O5W	1.0906 (4)	0.2219 (3)	0.6361 (2)	0.0417 (6)	
H5WA	1.202430	0.168969	0.610230	0.063*	
H5WB	1.061280	0.145700	0.690980	0.063*	
O6W	1.1261 (4)	0.2008 (3)	0.0149 (3)	0.0464 (7)	
H6WA	1.249795	0.169831	0.051457	0.070*	
H6WB	1.107965	0.109867	0.025091	0.070*	

### Tetraaquatrinitratoerbium/neodymium(0.359/0.641) dihydrate Atomic displacement parameters (Å^2^)

**Table:**

	*U*^11^	*U*^22^	*U*^33^	*U*^12^	*U*^13^	*U*^23^
Nd1	0.02410 (10)	0.02550 (10)	0.02437 (10)	−0.00855 (7)	0.00298 (6)	−0.00691 (7)
Er1	0.02410 (10)	0.02550 (10)	0.02437 (10)	−0.00855 (7)	0.00298 (6)	−0.00691 (7)
N1	0.0344 (16)	0.0303 (16)	0.0434 (18)	−0.0108 (13)	0.0029 (14)	−0.0039 (14)
N2	0.0388 (16)	0.0308 (16)	0.0351 (16)	−0.0129 (13)	0.0086 (13)	−0.0096 (13)
N3	0.0497 (19)	0.0408 (19)	0.0343 (17)	−0.0223 (16)	0.0000 (14)	−0.0048 (14)
O1	0.0450 (16)	0.0312 (14)	0.065 (2)	0.0025 (12)	0.0022 (14)	−0.0026 (13)
O2	0.0565 (17)	0.0453 (16)	0.0343 (15)	0.0001 (13)	0.0096 (13)	−0.0054 (12)
O3	0.0570 (18)	0.0501 (17)	0.0421 (16)	0.0003 (13)	−0.0021 (13)	−0.0180 (14)
O4	0.0664 (19)	0.0427 (16)	0.0442 (16)	−0.0208 (14)	0.0221 (14)	−0.0018 (13)
O5	0.0377 (15)	0.0444 (16)	0.077 (2)	−0.0102 (12)	0.0198 (14)	−0.0230 (15)
O6	0.0424 (14)	0.0334 (14)	0.0429 (15)	−0.0128 (11)	0.0039 (12)	−0.0023 (12)
O7	0.0483 (16)	0.0353 (14)	0.070 (2)	−0.0129 (12)	−0.0186 (15)	−0.0061 (14)
O8	0.0399 (15)	0.0501 (17)	0.0497 (17)	−0.0141 (13)	0.0000 (12)	−0.0187 (14)
O9	0.080 (2)	0.0497 (18)	0.0576 (19)	−0.0352 (16)	−0.0081 (16)	−0.0170 (15)
O1W	0.0702 (17)	0.0451 (16)	0.0291 (13)	−0.0351 (14)	0.0102 (12)	−0.0126 (12)
O2W	0.0446 (14)	0.0378 (14)	0.0289 (13)	−0.0148 (11)	−0.0013 (10)	−0.0075 (11)
O3W	0.0274 (12)	0.0334 (14)	0.0584 (17)	−0.0081 (10)	−0.0002 (11)	−0.0074 (12)
O4W	0.0419 (14)	0.0382 (14)	0.0369 (14)	−0.0152 (11)	0.0087 (11)	−0.0132 (11)
O5W	0.0456 (15)	0.0343 (14)	0.0417 (16)	−0.0138 (12)	−0.0002 (12)	−0.0101 (12)
O6W	0.0561 (17)	0.0349 (14)	0.0476 (17)	−0.0190 (13)	0.0074 (14)	−0.0116 (13)

### Tetraaquatrinitratoerbium/neodymium(0.359/0.641) dihydrate Geometric parameters (Å, º)

**Table:**

Nd1—N1	2.951 (3)	N2—O6	1.269 (3)
Nd1—N3	2.983 (3)	N3—O7	1.249 (3)
Nd1—O2	2.488 (2)	N3—O8	1.275 (4)
Nd1—O3	2.532 (3)	N3—O9	1.222 (4)
Nd1—O5	2.765 (3)	O1W—H1WA	0.9850
Nd1—O6	2.555 (2)	O1W—H1WB	0.9440
Nd1—O7	2.520 (3)	O2W—H2WA	0.9430
Nd1—O8	2.577 (3)	O2W—H2WB	0.9091
Nd1—O1W	2.375 (2)	O3W—H3WA	1.0640
Nd1—O2W	2.402 (2)	O3W—H3WB	1.0324
Nd1—O3W	2.397 (2)	O4W—H4WA	0.9341
Nd1—O4W	2.412 (2)	O4W—H4WB	0.9409
N1—O1	1.217 (3)	O5W—H5WA	0.8502
N1—O2	1.261 (4)	O5W—H5WB	0.8506
N1—O3	1.265 (4)	O6W—H6WA	0.8490
N2—O4	1.226 (3)	O6W—H6WB	0.8505
N2—O5	1.242 (3)		
			
N1—Nd1—N3	104.76 (9)	O3W—Nd1—O3	143.50 (9)
O2—Nd1—N1	25.00 (8)	O3W—Nd1—O5	68.96 (8)
O2—Nd1—N3	117.13 (10)	O3W—Nd1—O6	115.96 (8)
O2—Nd1—O3	50.23 (9)	O3W—Nd1—O7	72.11 (8)
O2—Nd1—O5	109.75 (8)	O3W—Nd1—O8	121.14 (8)
O2—Nd1—O6	68.28 (8)	O3W—Nd1—O2W	78.58 (9)
O2—Nd1—O7	139.95 (9)	O3W—Nd1—O4W	79.99 (8)
O2—Nd1—O8	92.68 (9)	O4W—Nd1—N1	92.62 (8)
O3—Nd1—N1	25.23 (8)	O4W—Nd1—N3	70.53 (8)
O3—Nd1—N3	89.96 (9)	O4W—Nd1—O2	116.81 (8)
O3—Nd1—O5	146.85 (9)	O4W—Nd1—O3	68.25 (8)
O3—Nd1—O6	100.48 (9)	O4W—Nd1—O5	131.29 (8)
O3—Nd1—O8	68.17 (9)	O4W—Nd1—O6	145.08 (9)
O5—Nd1—N1	130.47 (8)	O4W—Nd1—O7	71.11 (9)
O5—Nd1—N3	76.42 (8)	O4W—Nd1—O8	74.98 (8)
O6—Nd1—N1	84.09 (8)	O1—N1—Nd1	178.7 (3)
O6—Nd1—N3	76.73 (8)	O1—N1—O2	122.2 (3)
O6—Nd1—O5	47.29 (7)	O1—N1—O3	122.7 (3)
O6—Nd1—O8	70.21 (8)	O2—N1—Nd1	56.51 (16)
O7—Nd1—N1	129.06 (8)	O2—N1—O3	115.0 (3)
O7—Nd1—N3	24.43 (8)	O3—N1—Nd1	58.54 (16)
O7—Nd1—O3	111.76 (9)	O4—N2—O5	122.5 (3)
O7—Nd1—O5	64.22 (8)	O4—N2—O6	120.4 (3)
O7—Nd1—O6	84.12 (9)	O5—N2—O6	117.1 (3)
O7—Nd1—O8	49.59 (8)	O7—N3—Nd1	56.57 (19)
O8—Nd1—N1	79.85 (8)	O7—N3—O8	115.8 (3)
O8—Nd1—N3	25.18 (7)	O8—N3—Nd1	59.31 (17)
O8—Nd1—O5	89.76 (8)	O9—N3—Nd1	177.9 (3)
O1W—Nd1—N1	78.62 (8)	O9—N3—O7	122.3 (3)
O1W—Nd1—N3	142.15 (8)	O9—N3—O8	121.9 (3)
O1W—Nd1—O2	81.93 (9)	N1—O2—Nd1	98.50 (19)
O1W—Nd1—O3	77.75 (9)	N1—O3—Nd1	96.23 (19)
O1W—Nd1—O5	130.37 (8)	N2—O5—Nd1	93.0 (2)
O1W—Nd1—O6	140.38 (8)	N2—O6—Nd1	102.56 (19)
O1W—Nd1—O7	133.88 (9)	N3—O7—Nd1	99.0 (2)
O1W—Nd1—O8	139.03 (8)	N3—O8—Nd1	95.5 (2)
O1W—Nd1—O2W	71.50 (8)	Nd1—O1W—H1WA	114.3
O1W—Nd1—O3W	75.49 (8)	Nd1—O1W—H1WB	113.8
O1W—Nd1—O4W	71.66 (8)	H1WA—O1W—H1WB	100.2
O2W—Nd1—N1	92.63 (9)	Nd1—O2W—H2WA	115.7
O2W—Nd1—N3	144.04 (8)	Nd1—O2W—H2WB	112.7
O2W—Nd1—O2	70.02 (9)	H2WA—O2W—H2WB	99.7
O2W—Nd1—O3	115.48 (9)	Nd1—O3W—H3WA	130.7
O2W—Nd1—O5	68.45 (8)	Nd1—O3W—H3WB	126.0
O2W—Nd1—O6	74.03 (8)	H3WA—O3W—H3WB	103.3
O2W—Nd1—O7	130.56 (8)	Nd1—O4W—H4WA	111.7
O2W—Nd1—O8	143.99 (8)	Nd1—O4W—H4WB	113.3
O2W—Nd1—O4W	140.89 (8)	H4WA—O4W—H4WB	101.4
O3W—Nd1—N1	154.10 (9)	H5WA—O5W—H5WB	104.5
O3W—Nd1—N3	96.17 (9)	H6WA—O6W—H6WB	104.5
O3W—Nd1—O2	145.82 (10)		
			
O1—N1—O2—Nd1	−179.6 (3)	O5—N2—O6—Nd1	−0.9 (3)
O1—N1—O3—Nd1	179.6 (3)	O6—N2—O5—Nd1	0.8 (3)
O2—N1—O3—Nd1	−0.6 (3)	O7—N3—O8—Nd1	2.9 (3)
O3—N1—O2—Nd1	0.7 (3)	O8—N3—O7—Nd1	−3.0 (3)
O4—N2—O5—Nd1	−179.3 (3)	O9—N3—O7—Nd1	177.9 (3)
O4—N2—O6—Nd1	179.2 (3)	O9—N3—O8—Nd1	−178.0 (3)

### Tetraaquatrinitratoerbium/neodymium(0.359/0.641) dihydrate Hydrogen-bond geometry (Å, º)

**Table:**

*D*—H···*A*	*D*—H	H···*A*	*D*···*A*	*D*—H···*A*
O1*W*—H1*WA*···O6*W*^i^	0.99	1.84	2.744 (4)	150
O1*W*—H1*WB*···O5*W*^ii^	0.94	1.87	2.743 (4)	153
O2*W*—H2*WA*···O5*W*	0.94	1.79	2.678 (4)	155
O2*W*—H2*WB*···O5*W*^ii^	0.91	2.39	3.274 (4)	163
O2*W*—H2*WB*···O6^iii^	0.91	2.58	2.976 (4)	107
O3*W*—H3*WA*···O4^iv^	1.06	2.18	3.076 (4)	140
O3*W*—H3*WA*···O5^iv^	1.06	2.15	3.095 (4)	146
O3*W*—H3*WB*···O6^v^	1.03	2.35	3.206 (4)	140
O3*W*—H3*WB*···O8^v^	1.03	2.35	3.204 (4)	139
O4*W*—H4*WA*···O6*W*	0.93	1.85	2.727 (4)	156
O4*W*—H4*WB*···O6*W*^i^	0.94	2.39	3.312 (4)	166
O5*W*—H5*WA*···O4^v^	0.85	2.05	2.849 (4)	156
O5*W*—H5*WB*···O1^iii^	0.85	2.38	3.032 (4)	134
O5*W*—H5*WB*···O7^iv^	0.85	2.27	2.897 (4)	130
O6*W*—H6*WA*···O9^v^	0.85	1.99	2.834 (5)	176
O6*W*—H6*WB*···O1^vi^	0.85	2.19	2.922 (4)	144

Symmetry codes: (i) −*x*+2, −*y*+1, −*z*; (ii) −*x*+2, −*y*+1, −*z*+1; (iii) −*x*+1, −*y*+1, −*z*+1; (iv) −*x*+2, −*y*, −*z*+1; (v) *x*+1, *y*, *z*; (vi) *x*+1, *y*−1, *z*.
