# Supplementary figures and images for: Diadochic single crystal of an erbium–neodymium nitrate complex
Source: Acta Crystallogr E Crystallogr Commun. 2026 May 12;82(Pt 6):618–23. doi: 10.1107/S2056989026004615 (PMC13239009; doi:10.1107/S2056989026004615)

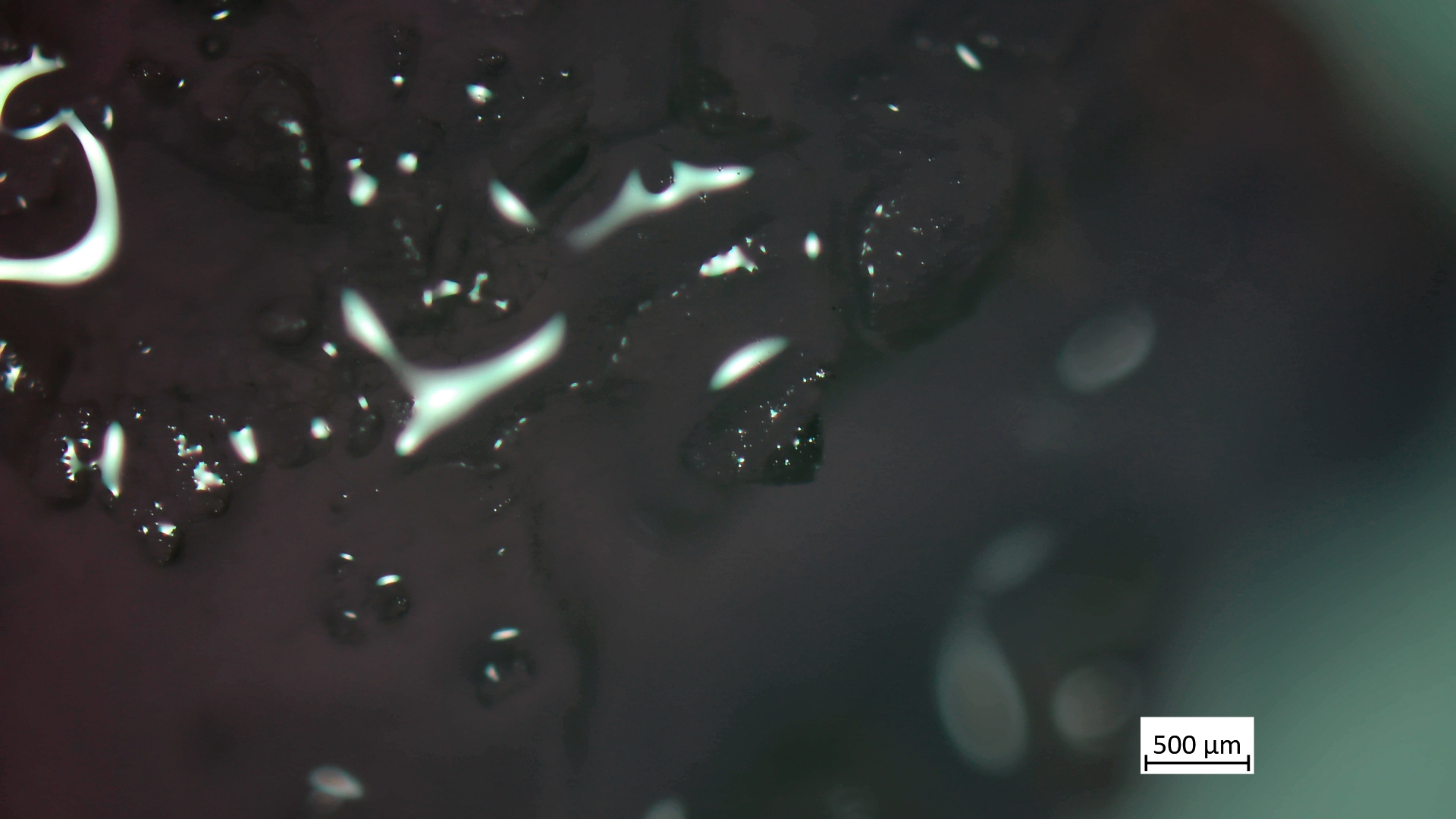

Supplement: Supplementary file 3 [file e-82-00618-sup4.jpg]

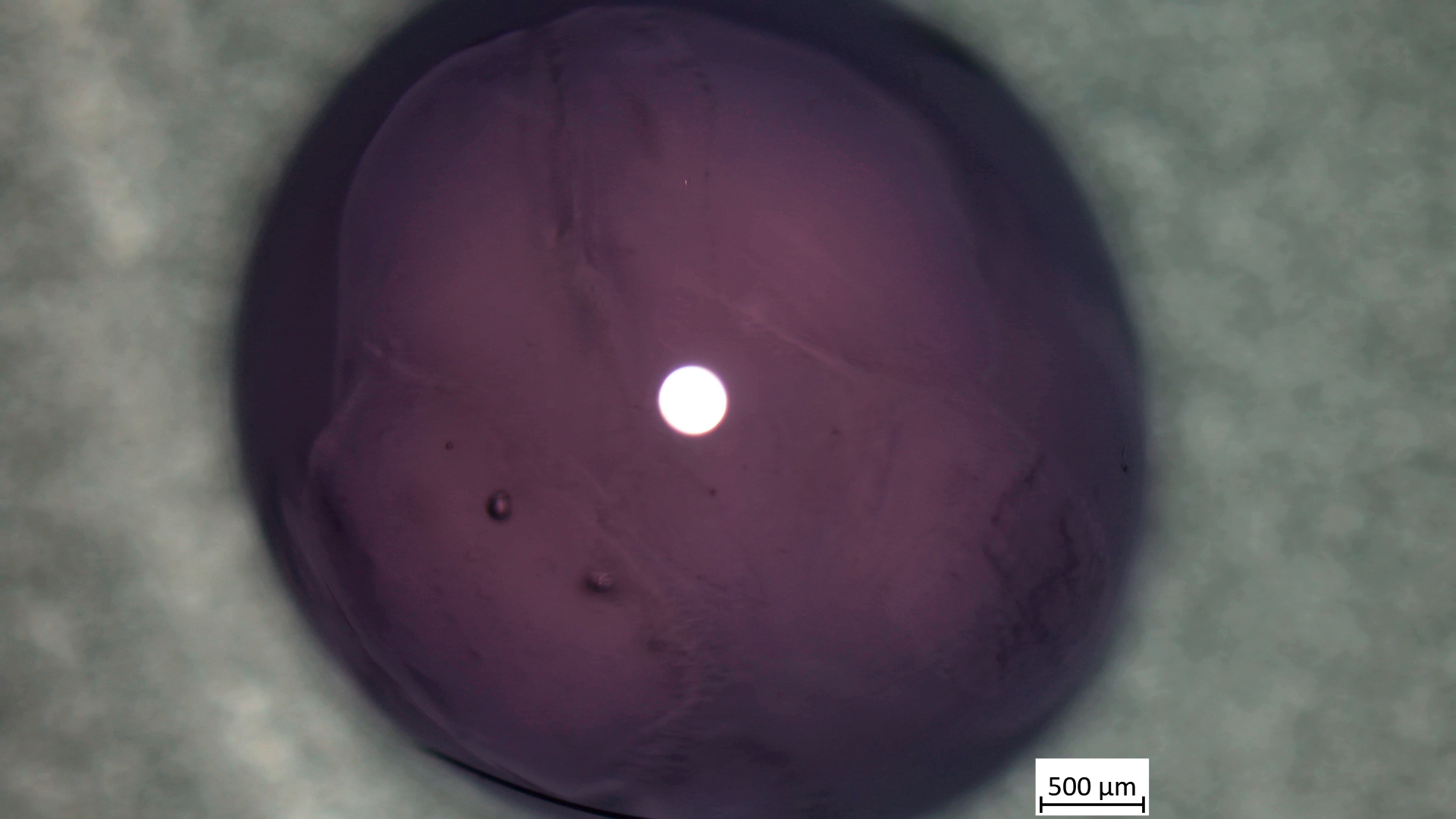

Supplement: Supplementary file 4 [file e-82-00618-sup5.jpg]
